# Transactions of the Chicago Gynæcological Society

**Published:** 1879-06

**Authors:** H. P. Merriman


### Society Reports.

### ARTICLE VIII.

### TRANSACTIONS OF THE CHICAGO GYNÆCOLOGICAL SOCIETY.

Seventh Meeting, April 25th, 1879. H. P. Merriman, M.D., in the chair.

Dr. DeLaskie Miller read a paper on "The Management of the Third Stage of Labor," published in the present number of the JOURNAL AND EXAMINER.

DR. ETHERIDGE thought that the so-called milk fever was probably not due to mammary congestion, but to the absorption of septic matter through the abrasions about the parturient canal.

DR. ROLER was in perfect accord with the views expressed by the reader of the paper. There should be no haste in the effort to secure contraction; this once attained, secured the woman against hæmorrhage. He never uses ergot during the second stage of labor, and in the third stage only when the placenta was in the act of being detached, or when inertia was present. Hæmorrhage had occurred very infrequently in his practice.

Concerning the use of disinfecting injections, the speaker found the practice objectionable, during the first twelve hours. Fresh wounds are capable of absorbing septic matter. After a time, wounds become glazed over and non-absorbing. There is danger of freshening these surfaces by the use of injections.

DR. DUDLEY thought it justifiable practice to inject the uterus itself with hot water in every case. It removed clots and tended to diminish after-pains. Hot water is especially useful in post partum hæmorrhage. On the other hand, he considered the use of Munsell's salt injudicious, and iodine much preferable, as being as efficient as a hæmostatic and safer. He mentioned an epidemic of puerperal fever occurring in Bellevue Hospital, when Dr. Perry demonstrated the great usefulness of injecting the uterus with hot carbolized water when there was a tendency to hæmorrhage, and a warm, carbolic injection repeated every four hours subsequently, to protect the canal from absorption of septic matter. The temperature was always greatly reduced by these injections.

Dr. FITCH thought that milk fever was due to mammary congestion, and that a free evacuation of the bowels was a neglected but excellent remedy. In the management of the third stage of labor, he exercised pressure freely, in a direction upward rather than downward, upon the body of the uterus. He also agitated the uterus to stimulate it to contraction ; he also made traction upon the cord, in the proper axis, for the same purpose. He usually inquired into the history of the previous labor of the patient, and governed himself accordingly. After labor he believed rest essential, and did not allow the clothing to be disturbed. He rarely introduced his hand into the uterus, and had never seen a case of so-called adherent placenta. He did not wash out the uterus or vagina, and had once known severe shock, fever and metritis to follow such injections.

DR. SAWYER alluded to the importance of what might be termed the fourth stage of labor, and spoke of Dewee's success in protecting the woman from after-pains by allowing the uterus to rest after delivery.

The speaker believed that the almost inevitable clot might be prevented by allowing the uterus time to recover itself after delivery ; by this means, the final tonic contraction occurs earlier and more strongly. After the lapse of 48 hours, he used vaginal irrigations of chlorinated soda.

DR. NELSON never left the uterus till the placenta was expelled, and made frequent examination for an hour after labor. He did not meddle unless dealing with a dilated womb or hæmorrhage ; then he introduced the hand and made compression. He gave ergot in abnormal cases, combining its use with quinine for some hours, or even days. He had known this course to prevent after-pains.

DR. JONES thought the views expressed were clear, succinct and eminently proper for the guidance of the accoucheur. He believed the normal state of the uterus just evacuated to be one of a more or less tonic contraction, which, by becoming complete, closed up the uterine sinuses. His plan of management of the third stage was influenced by the view. He never removed his hand from the uterine globe after the child was detached until the contraction was secured and became lasting. If the uterus was not plainly felt, he kneaded the organ until reaction was established, thus using pressure or agitation only when the organ was delinquent. In this manner he kept aware of the condition for at least an hour. He never gave ergot till after the birth of the child, and then with circumspection.

DR. MILLER concluded the discussion by enlarging upon the views expressed in the paper.

